# Comparing the effectiveness and safety of Sorafenib plus TACE with Apatinib plus TACE for treating patients with unresectable hepatocellular carcinoma: a multicentre propensity score matching study

**DOI:** 10.1186/s40644-023-00574-7

**Published:** 2023-05-30

**Authors:** Liang Yin, Kai-Cai Liu, Wei-Fu Lv, Dong Lu, Yu-Lin Tan, Guo-Xiang Wang, Jia-Ying Dai, Xian-Hai Zhu, Bo Jiang

**Affiliations:** 1 Department of Interventional Radiology, The First Affiliated Hospital of USTC, Division of Life Sciences and Medicine, University of Science and Technology of China, Hefei, 230001 Anhui China; 2 Infection Hospital, The First Affiliated Hospital of USTC, Division of Life Sciences and Medicine, University of Science and Technology of China, Hefei, 230000 Anhui China; 3 Department of Interventional Radiology, The First Affiliated Hospital of Bengbu Medical University, Bengbu, 233004 Anhui China; 4 Department of Interventional Radiology, The First Affiliated Hospital of Wannan Medical College, Wuhu, 241002 Anhui China; 5 Department of Interventional Radiology, Anqing Municipal Hospital, Anqing, 246003 Anhui China; 6 Department of Interventional Ultrasound, The Second Affiliated Hospital of Anhui Medical University, Hefei, 230000 China

**Keywords:** Hepatocellular carcinoma, Transarterial chemoembolization, Sorafenib, Apatinib, Clinical outcomes

## Abstract

**Objective:**

Local combined systemic therapy has been an important method for the treatment of unresectable hepatocellular carcinoma (HCC).The purpose of this study was to compare the effectiveness and safety of transarterial chemoembolization (TACE) plus Sorafenib versus TACE plus Apatinib for treating patients with unresectable HCC.

**Methods:**

The clinical data of patients with unresectable HCC who were treated with TACE plus Sorafenib or TACE plus Apatinib at 5 Chinese medical centers between January 2016 and December 2020 were retrospectively analyzed. Propensity score matching (PSM) was applied to reduce the bias from confounding factors.

**Results:**

A total of 380 patients were enrolled, of whom 129 cases were treated with TACE plus Sorafenib and 251 cases with TACE plus Apatinib. After the 1:1 PSM, 116 pairs of patients were involved in this study. The results showed that the PFS and OS in the TACE-Sorafenib group were significantly longer than those in the TACE-Apatinib group (PFS: 16.79 ± 6.45 vs. 14.76 ± 6.98 months, *P* = 0.049; OS: 20.66 ± 6.98 vs. 17.69 ± 6.72 months, *P* = 0.013). However, the ORR in the TACE-Apatinib group was markedly higher than that in the TACE-Sorafenib group (70.69% vs. 56.03%, *P* = 0.021). There were more patients with adverse events (AEs) in the TACE-Apatinib group than those in the TACE-Sorafenib group before dose adjustment (87 vs. 63, *P* = 0.001); however, the number of patients who suffered from AEs was not significantly different between the two groups after the dose adjustment (62 vs. 55, *P* = 0.148). No treatment-related death was found in the two groups. Subgroup analysis revealed that patients with unresectable HCC could better benefit from regular doses than reduced doses (Sorafenib, 22.59 vs. 18.02, *P* < 0.001; Apatinib, 19.75 vs. 16.86, *P* = 0.005).

**Conclusion:**

TACE plus either Sorafenib or Apatinib could effectively treat patients with unresectable HCC, the safety of TACE plus Sorafenib was better. and the ORR of TACE plus Apatinib was higher.

## Introduction

Hepatocellular carcinoma (HCC) is one of the most common malignant tumors in the digestive system, and surgical resection is the preferred treatment for HCC. However, early diagnosis of HCC is still a main challenge, and only a very low percentage of patients could be treated by surgical resection, while their prognosis is extremely poor and the survival time is very short [1, 2]. The sensitivity of HCC to intravenous chemotherapy is very poor, which could involve various adverse events (AEs) [3]. Compared with systemic chemotherapy, transarterial chemoembolization (TACE) could directly increase the drug concentration in local tumor tissues, thereby exerting the killing effects more effectively; in addition, TACE could block the tumor blood flow, induce necrosis and apoptosis of tumor cells, and effectively control tumor progression. Therefore, TACE could be one of the options for patients who are not treated surgically [4–7]. The expressions of various factors, including vascular endothelial growth factor (VEGF) and matrix metallopeptidase 9 (MMP-9), are upregulated in the ischemic and hypoxic environments, following TACE, which could be one of the mechanisms inducing tumor recurrence and metastasis [8, 9]. Previous studies have demonstrated that VEGF could stimulate the generation of matrix metalloproteinases (MMPs) by endothelial cells [8, 10]; therefore, anti-tumor drugs targeting VEGF signaling pathway have significant effects on tumor-feeding arteries [11]. Thus, the combination of VEGF inhibitors following TACE could, theoretically, regulate the expressions of cancer-promoting factors, e.g. VEGF and MMP-9, inhibit angiogenesis of tumors, and eventually reduce the possibility of tumor recurrence and metastasis.

Sorafenib is a small-molecule multi-kinase inhibitor that is clinically used for the treatment of HCC. It inhibits kinases such as Raf kinase, vascular endothelial growth factor receptor (VEGFR), and platelet-derived growth factor receptor (PDGFR)-β tyrosinek inases. It also inhibits tumor-cell proliferation and angiogenesis and increases the rate of apoptosis in a wide range of tumor models [12, 13]. Clinical studies have demonstrated that molecular targeted therapies have not only specific anti-tumor effects, but also lower systemic side effects than conventional chemotherapeutic drugs [14]. A clinical study on HCC patients conducted by Cheng et al. [15] showed that Sorafenib could significantly improve the overall survival (OS) of HCC patients. Studies performed in other countries (except for China) also revealed that Sorafenib could improve the survival time of patients with intermediate and advanced HCC. However, there are certain limitations in the clinical application of Sorafenib in China due to several causes, including being clinically cost-effective.

Apatinib is a novel small-molecule tyrosine kinase inhibitor that was independently developed by Chinese scholars. The major target of Apatinib is the VEGFR-2/kinase insert domain receptor (KDR), selectively inhibiting the activities of VEGFR-2, as well as blocking the signal transduction of the binding of VEGF to receptors, effectively inhibiting the cellular processes (proliferation and migration of endothelial and tumor epithelial cells), thereby inhibiting tumor angiogenesis and exerting anti-tumor effects [16, 17]. The phase II clinical trials [18] on treating HCC by Apatinib showed that the time to tumor progression was significantly longer than in the SHARP trial(4.2 m vs 2.8 m),A phase III clinical study on sorafenib as a first-line treatment for patients with HCC [19]. Other clinical studies also demonstrated that treatment with Apatinib could remarkably increase the objective response rate (ORR) and disease control rate (DCR), while the incidence of AEs was reduced [20, 21]. Therefore, TACE plus Apatinib could exert more comprehensive anti-tumor effects, thereby providing a new choice for improving the clinical efficacy, particularly for HCC. As no prospective or retrospective study comparing TACE plus Sorafenib with TACE plus Apatinib has been conducted yet, the present study aimed to compare the effectiveness and safety of TACE plus Sorafenib versus TACE plus Apatinib for treating advanced HCC patients.

## Patients and methods

### Study design

In this multi-center retrospective study, all patients who were diagnosed with HCC and were treated with TACE plus Sorafenib or TACE plus Apatinib at 5 Chinese medical centers in Anhui province (the First Affiliated Hospital of University of Science and Technology of China, the First Affiliated Hospital of Bengbu Medical College, the First Affiliated Hospital of Wannan Medical College, Anqing Municipal Hospital, and the Second Affiliated Hospital of Anhui Medical University) between January 2016 and December 2020 were included. This study was approved by the Ethics Committees of all the above-mentioned medical centers, and it was performed according to the Declaration of Helsinki. As this was a retrospective study, informed consent was waived by the Ethics Committees. All patients’ data were kept confidential. The inclusion criteria were as follows: 1) patients who were clinically diagnosed with primary HCC; 2) patients in Barcelona Clinic Liver Cancer (BCLC) stage B/C; 3) Child–Pugh class A/B; 4) the existence of complete imaging data (computed tomography (CT)/magnetic resonance imaging (MRI)) before surgery and during follow-up; 3) the East Cooperative Oncology Group (ECOG) performance status (PS) score of 0 or 1; 6) patients without contraindication to TACE, and those who received targeted therapy after TACE; and 7) the duration of undergoing targeted therapy and expected survival time would be > 3 months. The exclusion criteria were as follows: 1) patients who had been treated with other therapeutic methods, including systemic chemotherapy, immunotherapy, and radiofrequency ablation (RFA); 2) patients with other severe diseases (Combined with other tumors, or cardiovascular and cerebrovascular diseases, etc.); 3) patients with severe coagulation disorders (Patients with prothrombin time > 18 s or a tendency to bleed); and 4) patients who suspended the therapy for more than 1 month or changed the targeted therapeutic drugs within 1 month during follow-up.

### Treatment

TACE was performed by experienced interventional radiologists who had at least 5 years of experience in TACE. The modified Seldinger technique was adopted to puncture the right femoral artery, and a 5F femoral artery sheath was placed. A 5F-RH catheter was delivered to the celiac trunk under the guiding of 0.032 in super-smooth guidewire for angiography, which clarified the tumor site and blood supply, as well as the existence of hepatic arterioportal fistulas and hepatic arteriovenous fistulas. Afterward, the coaxial microtube method was adopted for the delivery of the microcatheter, and chemotherapeutic drugs (e.g., 3–20 mg Lipiodol, 20–40 mg Epirubicin, and 150 mg Oxaliplatin) were injected into the tumor-feeding arteries according to the sizes of tumors. After the tumor vascular bed was saturated and the blood flow in portal vein branches adjacent to the tumor were slowed down, then, polyvinyl alcohol (PVA) particles (150–350 or 350–560 μm, and 560–710 μm) were infused slowly until the blood flow was completely blocked. The sizes of PVA particles were selected according to the superselective catheterization of hepatic artery and sizes of tumors.

### Administration of Apatinib or Sorafenib

Regarding patients in the TACE-Apatinib group and TACE-Sorafenib group, Apatinib with the initial dose of 500 mg/d or Sorafenib with the initial dose of 400 mg/bid was orally administered at 3–5 days after TACE, respectively. If grade 3–4 drug-related AEs occurred, according to the Common Terminology Criteria for Adverse Events (ver. 4.0; CTCAE 4.0), the doses of oral Apatinib and Sorafenib were adjusted to 250 mg/d and 200 mg/bid, respectively, or the Apatinib and Sorafenib treatments could be discontinued for several days. When AEs improved, the patients were suggested to restore the previous doses until disease progression, death, or one of the following items requiring treatment to be discontinued: AEs requiring discontinuation of treatment, ECOG-PS score worsened to 4 points, deterioration of liver function, or a patient’s clinical requirements.

### Follow-up

Follow-up was undertaken for all patients at 1 month after TACE, and repeated TACE could be performed according to the conditions of lesions, with the interval of 1.5–2 months. Patients with complete response (CR) were followed up every 3 months, in which evaluation of liver function, alpha-fetoprotein (AFP) screening, and contrast-enhanced CT or MRI were carried out. For patients who used Sorafenib or Apatinib, the liver and renal functions, blood circulation, and coagulation function were assessed every month. Patients were treated with continuous targeted therapy until intolerable AEs occurred, or tumor progressed, and patients could not benefit from the treatment according to clinical decisions. All patients were followed up until December 30, 2021.

### Tumor responses and safety evaluation

Regarding patients in the two groups, the tumor responses were evaluated every 8 weeks, according to the modified Response Evaluation Criteria in Solid Tumors (mRECIST) and contrast-enhanced CT(loversol) or MR(Primovist) images. A complete response (CR) was defined as the disappearance of any arterial enhancement in the target tumor, a partial response (PR) was defined as over 30% decrease in the sum of the diameters of viable lesions, progressive disease (PD) was defined as over 20% increase in the sum of the diameters of viable lesions, and a stable disease (SD) was defined as any cases with nonPR or nonPD. An objective response rate (ORR) was defined as the percentage of patients achieving either CR or PR, and disease control rate (DCR) as the percentage of patients achieving CR, PR, or SD. The tumor response of the target lesion was independently evaluated by two Senior radiologists (over 10 years of experience in radiology,) who were blinded to each other's conclusions.Disagreements were resolved by discussion until consensus could be achieved. The best overall response during the treatment was considered as the final response. The treatment-related AEs were classified according to the CTCAE 4.0. The vital signs and AEs were monitored throughout the study. The safety evaluation was mainly based on the occurrence, frequency, and severity of AEs. The safety was evaluated by the vital signs, results of physical examinations, data of clinical examinations, results of laboratorial examinations, and AEs. The safety of the two groups (TACE-Sorafenib and TACE-Apatinib groups) was evaluated, which included the proportion of patients who discontinued targeted therapy and patients who received a reduced dosage due to AEs.

### Statistical analysis

The SPSS 22.0 software (IBM Corp., Armonk, NY, USA) was used to carry out statistical analysis. A logistic regression model was utilized to calculate the propensity scores of the patients, in which the propensity score matching (PSM) was accordingly performed. The covariates included in the analysis were gender, age, viral hepatitis, Child–Pugh score, serum AFP level, BCLC stage, ECOG-PS score, number of nodules, tumor size, liver function, vascular invasion, extrahepatic metastasis, frequency of undergoing TACE, and history of anti-tumor therapy. The 1:1 nearest neighbor matching was adopted. The Fisher’s exact test or χ^2^ test, as well as the Student’s t-test were utilized to compare differences in the above-mentioned characteristics between the two groups. The Kaplan–Meier method was adopted to estimate the rates of progression-free survival (PFS) and OS. *P* < 0.05 was considered statistically significant.

## Results

### Patients’ demographic and clinical characteristics at baseline

**Fig. 1:**
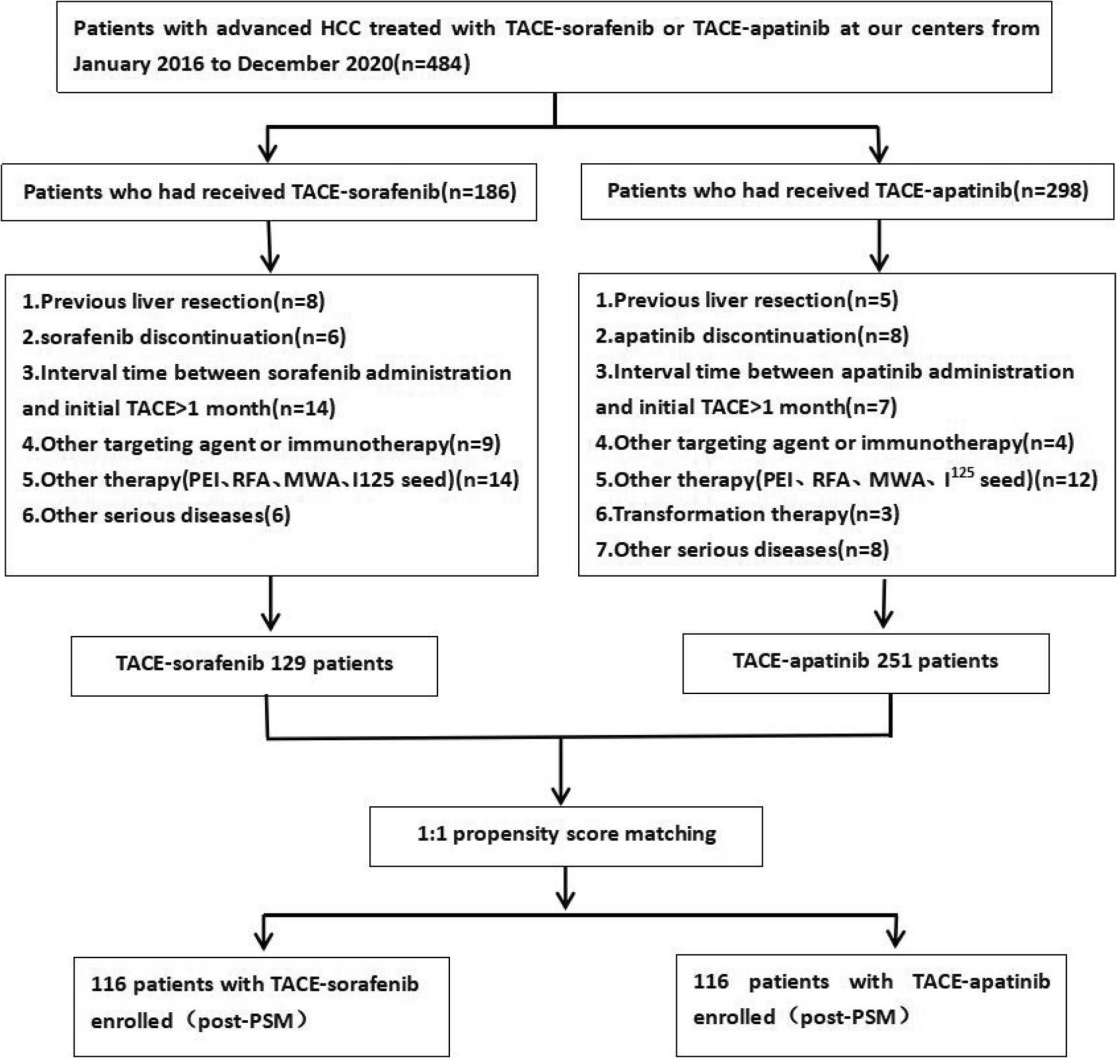
Patient recruitment flowchart

Totally, 484 patients with unresectable HCC met the inclusion criteria, of whom 104 cases were excluded according to the exclusion criteria. Finally, the data of 380 patients were included in the analysis, of whom 129 cases were treated with TACE plus Sorafenib and 251 with TACE plus Apatinib. After the PSM, 116 pairs of patients were matched (shown in Fig. 1). The serum FAP level, the number of nodules, tumor size, liver function, extrahepatic metastasis, and history of anti-tumor therapy were all significantly different between the two groups before PSM analysis. In the PSM cohort,, the baseline characteristics were not significantly different between the two groups (shown in Table 1).

**Table 1:** The baseline characteristics between the two groups before and after PSM analysis

Variables	Before PSM			After PSM		
	TACE + Sorafenib (*n* = 129)	TACE + aptinib (*n* = 251)	*p*	TACE + Sorafenib (*n* = 116)	TACE + aptinib (*n* = 116)	*p*
Sex			0.201			0.082
Male	112	205		101	91	
Female	17	46		15	25	
Age (y)	55.04 ± 12.05	55.38 ± 11.77				
Viral hepatitis						0.120
Yes	100	203	0.441	94	84	
No	29	48		22	32	
Child–Pugh			0.992			0.293
A	76	148		65	57	
B	53	103		51	59	
AFP			0.785			0.427
≤ 400 ng/ml	81	154		68	62	
> 400 ng/ml	48	97		48	54	
ECOG PS			0.262			0.351
0	81	172		71	64	
1/2	48	79		45	52	
Number of nodules			0.222			0.232
≤ 3	64	108		54	45	
> 3	65	143		62	71	
Tumor size			0.005			0.089
≤ 5 cm	35	105		30	42	
> 5 cm	94	146		86	74	
Liver cirrhosis			0.081			0.127
Yes	79	176		78	66	
No	50	75		48	60	
MVI			0.833			0.511
Yes	71	141		60	55	
No	58	110		56	61	
Extrahepatic metastasis			0.076			0.280
Yes	23	65		31	24	
No	106	186		85	92	
Number of TACE			0.255			0.419
≤ 3	52	117		48	42	
> 3	77	135		68	74	

*TACE* Transcatheter arterial chemoembolization, *PSM* Propensity score matching, *ECOG PS* Eastern Cooperative Oncology Group Performance Status, *AFP* Alpha-fetoprotein, *MVI* Microvascular invasion

### Therapeutic effects

Among 116 patients who were treated with TACE plus Sorafenib, CR, PR, SD, and PD were observed in 6, 59, 37, and 14 patients after 8 weeks, respectively, according to the mRECIST criteria. The ORR and DCR were 56.03% and 87.93%, respectively. Among 116 patients who were treated with TACE plus Apatinib, CR, PR, SD, and DP were observed in 9, 73, 26, and 8 patients, respectively. The ORR and DCR were 70.69% and 93.10%, respectively. The ORR was significantly different between the two groups (*P* = 0.021), while the DCR was not significantly different (shown in Table 2).

**Table 2:** Comparison of the short-term effects between the two groups

	Complete response (CR)	Partial response (PR)	Stable disease (SD)	Progressive disease (PD)	Objective response rate (ORR)	Disease control rate (DCR)
TACE + Sorafenib (*n* = 116)	6(5.17%)	59(50.86%)	37(31.90%)	14(12.07%)	65(56.03%)	102(87.93%)
TACE + aptinib (*n* = 116)	9(7.76%)	73(62.93%)	26(22.41%)	8(6.90%)	82(70.69%)	108(93.10%)
X^2^					5.366	1.808
*P*					0.021	0.179

### PFS and OS

**Fig. 2:**
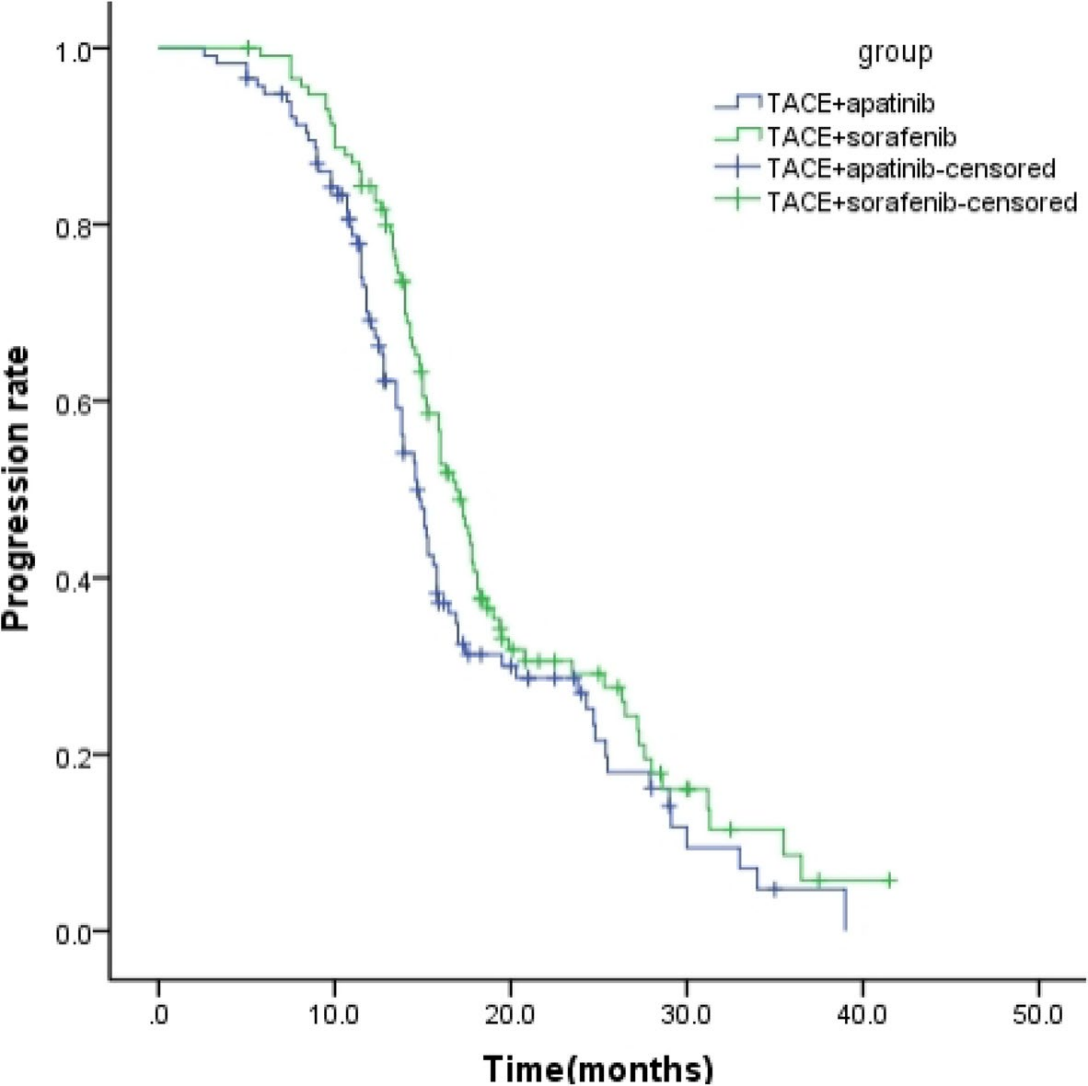
Kaplan–Meier curves of time to progression for patients with advanced hepatocellular carcinoma who received the treatment of TACE–apatinib or TACE-Sorafenib after propensity score matching

**Fig. 3:**
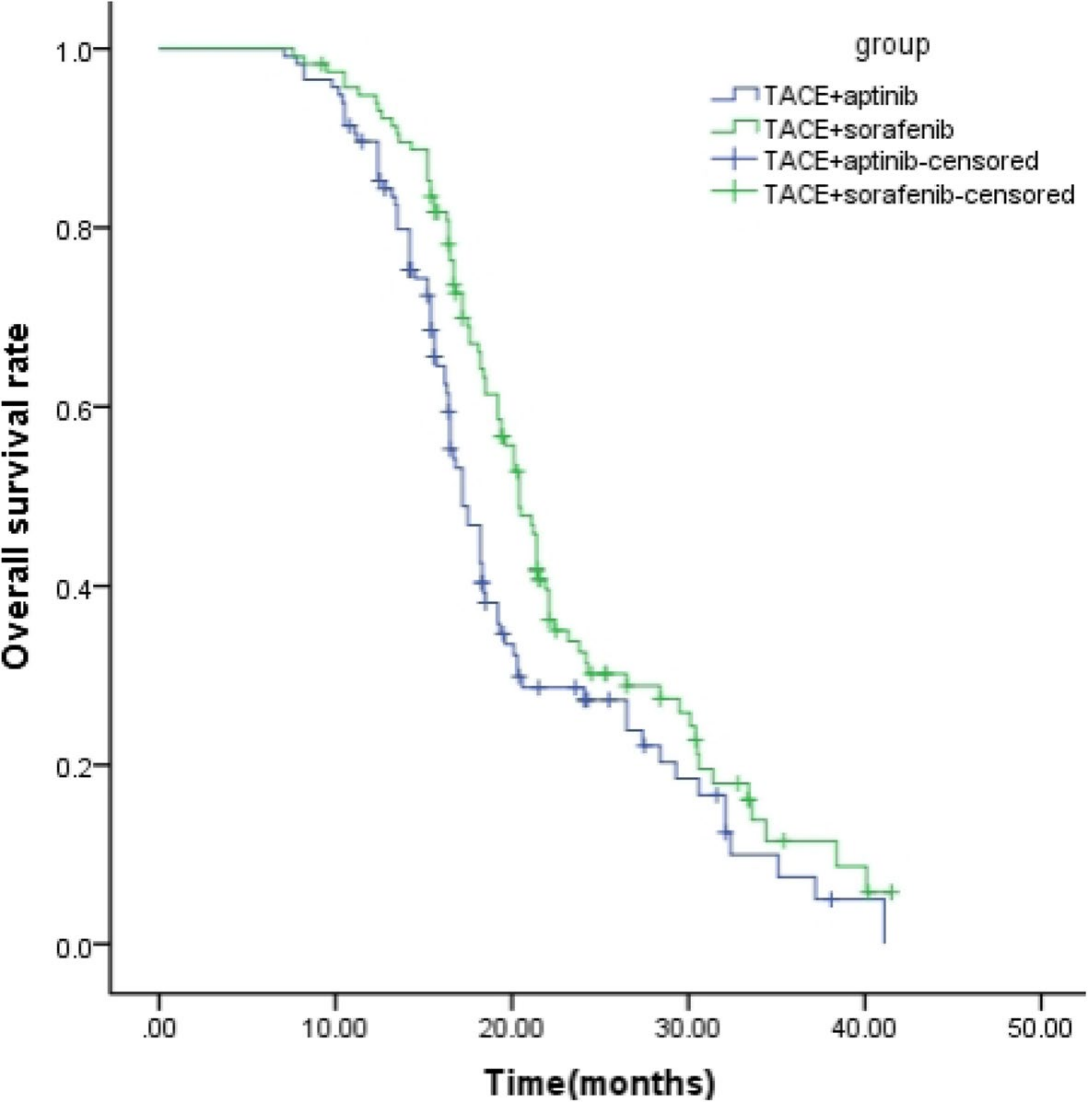
Kaplan–Meier curves of time to overall survival for patients with advanced hepatocellular carcinoma who received the treatment of TACE–apatinib or TACE-Sorafenib after propensity score matching

The follow-up period ranged from 3.5 to 37.3 months, with a mean of 17.3 months. The median OS time was 18.8 months (95% confidence interval, 16.0–22.4 months).The median PFS was 16.79 ± 6.45 months (95% confidence interval [CI]: 16.09–18.51 months) in the TACE-Sorafenib group, which was significantly longer than that in the TACE-Apatinib group (14.76 ± 6.98 months; 95% CI: 13.53–15.87 months) (*P* = 0.049, Fig. 2). The median OS was 17.69 ± 6.72 months (95%CI: 15.91–18.49 months) and 20.66 ± 6.98 months (95%CI: 19.17–21.63 months) in the TACE-Apatinib and TACE-Sorafenib groups, respectively. The 1- and 2-year survival rates were 93.97% and 23.28% in the TACE-Sorafenib group, and 87.93% and 18.97% in the TACE-Apatinib group, and the difference was statistically significant between the two groups (*P* = 0.013, Fig. 3).

### Comparison of treatment effectiveness

Among 116 patients in the TACE-Apatinib group, the dose of Apatinib was not adjusted in 60 (60/116, 51.72%) patients during the treatment, in which the maintenance dose was 500 mg/d; however, the dose of Apatinib was adjusted in 56 (56/116, 48.28%) patients, mainly due to the occurrence of hand-foot syndrome (HFS) (25/56, 44.64%), abdominal pain (5/56, 8.93%), and hematological toxicity (5/56, 8.93%). Eight of the patients were found with drug discontinuation, due to proteinuria (7/8, 87.5%) and gastrointestinal bleeding (1/8, 12.5%). Among 116 patients in the TACE-Sorafenib group, the dose of Sorafenib was not adjusted in 79 (79/116, 68.10%) patients during the treatment, in which the maintenance dose was 800 mg/d; however, the dose of Sorafenib was adjusted in 37 patients, mainly due to the occurrence of HFS (19/37, 51.35%) and proteinuria (8/37, 21.62%), while there was no patient with drug discontinuation. There were more patients with AEs in the TACE-Apatinib group than those in the TACE-Sorafenib group before dose adjustment (87 vs. 63; χ^2^ = 10.86, *P* = 0.001); however, the difference in the incidence of AEs was not statistically significant between the two groups after the dose adjustment (62 vs. 55, χ^2^ = 0.845, *P* = 0.358). The incidence of AEs was significantly different in the TACE-Apatinib group before and after the dose adjustment (χ^2^ = 11.72, *P* < 0.01), while there was no significant difference in the TACE-Sorafenib group before and after the dose adjustment (χ^2^ = 1.104, *P* = 0.293) (shown in Table 3).

**Table 3:** Adverse events in the normal-dose and the reduced-dose after propensity score matching in the TACE–apatinib group and TACE-Sorafenib group

Adverse events	TACE-Sorafenib group						TACE-Apatinib group						*P*			
	Normal-dose			Reduced-dose			Normal-dose			Reduced-dose						
	All grade(n)	grade1/2(n)	grade3/4(n)	All grade(n)	grade1/2(n)	grade3/4(n)	All grade(n)	1/2grade(n)	3/4grade(n)	All grade(n)	1/2grade(n)	3/4grade(n)	P^1^	P^2^	P^3^	P^4^
Hand and foot syndrome	61(52.59%)	42(36.21%)	19(16.38%)	50(43.10%)	42(36.21%)	8(6.90%)	65(56.03%)	40(34.48%)	25(19.84%)	60(51.72%)	48(41.38%)	12(10.34%)	0.598	0.189	0.064	0.023
Hypertension	31(26.72%)	20(17.24)	11(9.48%)	24(20.69%)	19(16.38)	5(4.31%)	53(45.69%)	38(32.76%)	15(12.93%)	40(34.48%)	34(29.31%)	6(5.17%)	0.003	0.081	0.003	0.028
Proteinuria	20(17.24%)	12(10.34%)	8(6.90%)	15(12.93%)	11(9.48%)	4(3.45%)	28(18.10%)	18(15.52%)	10(8.62%)	16(13.79%)	14(12.07%)	2(1.72%)	0.195	0.847	0.359	0.044
Alopecia	6(5.17%)	4(3.45%)	2(1.72%)	4(3.45%)	3(2.59%)	1(0.86%)	5(4.31%)	3(2.59%)	2(1.72%)	4(3.45%)	3(2.59%)	1(0.86%)	0.757	1.000	0.778	0.635
Fatigue	22(18.5%)	19(16.38%)	3(2.59%)	18(15.52%)	17(14.66%)	1(0.86%)	28(24.14%)	23(19.83%)	5(4.31%)	19(16.38%)	17(14.66%)	2(1.72%)	0.338	0.858	0.398	0.488
Decreased appetite	24(20.69%)	16(13.79%)	8(6.90%)	17(14.66%)	15(12.93%)	2(1.72%)	27(23.28%)	20(17.24)	7(6.03%)	13(11.21%)	10(8.62%)	3(2.59%)	0.634	0.434	0.113	0.845
Diarrhea	34(29.31%)	30(25.86%)	4(3.45%)	21(18.10%)	21(18.10%)	0	17(14.66%)	15(12.93%)	2(1.72%)	13(11.21%)	12(10.34%)	1(0.86%)	0.002	0.137	0.111	0.713
Oral mucositis	21(18.10%)	19(16.38%)	2(1.72%)	15(12.93%)	15(12.93%)	0	36(31.03%)	32(27.59%)	4(3.45%)	28(24.14%)	25(21.55%)	3(2.59%)	0.022	0.028	0.218	0.959
hoarseness	7(6.03%)	6(5.17%)	1(0.86%)	4(3.45%)	4(3.45%)	0	10(8.62%)	6(5.17%)	4(3.45%)	7(6.03%)	6(5.17%)	1(0.86%)	0.449	0.354	0.428	0.252
Abdominal pain	27(23.28%)	24(20.69%)	3(2.59%)	21(18.10%)	20(17.24%)	1(0.86%)	33(28.45%)	28(24.14%)	5(4.31%)	27(23.28%)	25(21.55%)	2(1.72%)	0.368	0.331	0.429	0.353
Hematotoxicity	15(12.93%)	13(11.21%)	2(1.72%)	10(8.62%)	9(7.76%)	1(0.86%)	21(16.67%)	16(13.79%)	5(4.31%)	15(12.93%)	13(11.21%)	2(1.72%)	0.277	0.289	0.802	0.434
Rash	24(20.68%)	20(17.24%)	4(3.45%)	15(12.93%)	13(11.21%)	2(1.72%)	26(22.41%)	21(18.10%)	5(4.31%)	20(17.24%)	17(14.66%)	3(2.59%)	0.749	0.359	0.779	0.707
Vomiting	12(10.34%)	10(8.62%)	2(1.72%)	8(6.90%)	7(6.03%)	1(0.86%)	15(12.93%)	12(10.34%)	3(2.59%)	12(10.34%)	10(8.62%)	2(1.72%)	0.539	0.349	0.825	0.798
constipation	4(3.45%)	2(1.72%)	2(1.72%)	2(1.72%)	2(1.72%)	0	3(2.59%)	2(1.72%)	1(0.86%)	1(0.86%)	1(0.86%)	0	0.701	0.561	0.221	0.386
headache	11(9.48%)	9(7.76%)	2(1.72%)	7(6.03%)	7(6.03%)	0	15(12.93%)	12(10.34%)	3(2.59%)	10(8.62%)	9(7.76%)	1(0.86%)	0.405	0.450	0.231	0.504
Liver dysfunction	4(3.45%)	3(2.59%)	1(0.86%)	2(1.72%)	2(1.72%)	0	6(5.17%)	4(3.45%)	2(1.72%)	2(1.72%)	2(1.72%)	0	0.518	1.000	0.439	0.221
Gastrointestinal hemorrhage	2(1.72%)	2(1.72%)	0	0	0	0	3(2.59%)	2(1.72%)	1(0.86%)	1(0.86%)	1(0.86%)	0	0.651	0.316	/	0.505
number of cases	63(54.31%)			55(47.41%)			87(75.00%)			62(53.45%)			0.001	0.358	0.293	0.001

P^1^: comparison of the incidence of adverse reaction rate between the two groups at normal-dose

P^2^: comparison of the incidence of adverse reaction rate between the two groups at reduced-dose

P^3^: comparison of adverse reaction rate between normal-dose and reduced-dose in TACE sorafenib group

P^4^: comparison of adverse reaction rate between normal-dose and reduced-dose in TACE-apatinib group

Note: some patients have a variety of adverse reactions, so the total number of adverse reactions is greater than the number of cases

### Subgroup analysis with or without dose adjustment

**Fig. 4:**
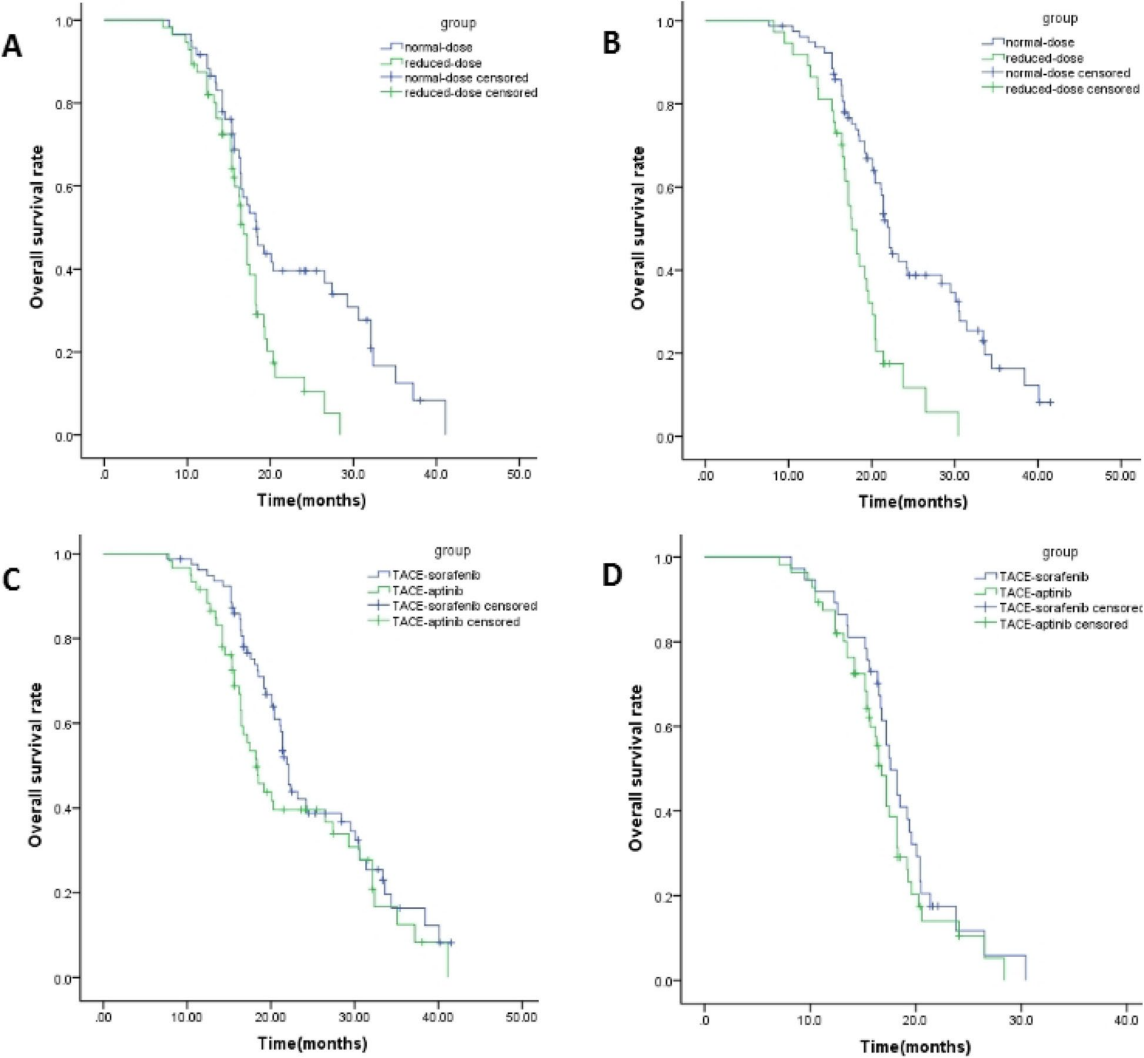
Kaplan–Meier survival curves. **A** OS between normal-dose and reduced-dose subgroup in the TACE-apatinib; **B** OS between normal-dose and reduced-dose subgroup in the TACE-sorafenib; **C** OS in the normal-dose subgroup between TACE–apatinib and TACE-Sorafenib; **D** OS in the reduced-dose subgroup between TACE–apatinib and TACE-Sorafenib

Subgroup analysis was performed to further explore the influences of different dosages of drugs on the survival time of patients. The results showed that for either TACE-Sorafenib or TACE-Apatinib, the survival time of patients was significantly longer when treated with the regular dose than the reduced dose (Apatinib, 19.75 vs. 16.86, *P* = 0.005, Fig. 4A; Sorafenib, 22.59 vs. 18.02, *P* < 0.001, Fig. 4B). The survival time of patients treated with Sorafenib and Apatinib was not significantly different between the regular dose subgroup (22.59 vs. 19.75, *P* = 0.118, Fig. 4C) and the reduced dose subgroup (18.02 vs. 16.86, *P* = 0.242, Fig. 4D).

## Discussion

In recent years, the incidence of HCC has increased gradually in China, accounting for about 50% of HCC patients in the world. About 70–85% of patients are at intermediate or advanced stage during diagnosis, and thus, they have already lost the chance of surgical treatment [22]. According to the BCLC staging and treatment guidelines for HCC, as well as the Chinese standards for diagnosis and treatment of primary HCC, TACE-based combination therapies are the standard treatments for patients with intermediate or advanced HCC [23, 24]. TACE mainly reduces or blocks the tumor-feeding arteries to induce the hypoxia and necrosis in multicellular tumor spheroids; however, TACE could also simultaneously induce VEGF expression, resulting in elevating VEGF expression in residual tumors, thereby promoting the metastasis and cancer recurrence [25, 26]. Therefore, inhibiting the VEGFs could block or reduce tumor angiogenesis, and inhibit the recurrence and metastasis of cancer cells. The VEGF family of growth factors and its receptors constitute the most important signaling pathways in tumor angiogenesis; therefore, TACE in combination with VEGF inhibitors could be used for further treatment of patients with intermediate or advanced HCC.

Sorafenib is the first agent that could improve the survival of patients with advanced HCC. Numerous clinical studies have reported promising clinical efficacy of Sorafenib for HCC patients, especially for patients with intermediate or advanced HCC [15, 27]. Multiple phase II/III clinical trials have already demonstrated that Apatinib possesses different clinical efficacies for various solid tumors, including gastric cancer, breast cancer, and primary HCC [28, 29]. However, to date, only few studies have compared the efficacy of TACE plus Sorafenib with that of TACE plus Apatinib for patients with intermediate or advanced HCC, and the clinical data are therefore limited.

In the present retrospective study, a total of 232 patients who had received TACE treatment were assigned into the TACE-Sorafenib group and TACE-Apatinib group. The short-term efficacy after 8 weeks of treatment was evaluated according to the mRECIST criteria. The results showed that the ORR was 70.69% in the TACE-Apatinib group, which was significantly higher than 56.03% in the TACE-Sorafenib group (*P* = 0.021); however, the DCR was not significantly different between the two groups (93.10% vs. 87.93%, *P* = 0.179). We speculated that the higher ORR in the TACE-Apatinib group could be associated with the fact that the affinity of Apatinib to VEGFR is tenfold of the affinity of Sorafenib to VEGFR, which could exert the effects through the VEGF pathway, inhibit the intracellular adenosine triphosphoric acid binding site of the VEGFR-2, reduce the tumor microvessel density, and promote the cell apoptosis [30, 31]. These could also be the causes of undergoing rapid transformational therapy in 3 patients in the TACE-Apatinib group that could be discovered during the screening process.

The patients’ median PFS was 17.01 and 14.76 months in the TACE-Sorafenib and TACE-Apatinib groups, respectively, both of which were longer than 3 months that was reported by Liu et al. for patients with unresectable HCC who were treated with TACE alone[3]. In addition, there was a significant difference in PFS between the TACE-Sorafenib and TACE-Apatinib groups. At the end of the follow-up, the median OS in the TACE-Sorafenib group was significantly longer than that in the TACE-Apatinib group (20.66 ± 6.98 vs. 17.69 ± 6.72 months, *P* = 0.013). The findings of the present study were in agreement with those reported previously [32], which demonstrated that both treatments could provide short-term and long-term effectiveness for patients with advanced HCC. The above-mentioned results indicated that combination of targeted therapy with TACE could result in higher ORR and DCR, and increase patients’ PFS. Besides, the higher effectiveness of TACE-Sorafenib compared with TACE-Apatinib for patients with advanced HCC was proved.

In the present study, AEs in the TACE-Apatinib group mainly included proteinuria, hypertension, weakness, and HFS, which were in agreement with previous studies [33–35]. Besides, AEs in the TACE-Sorafenib group mainly included diarrhea and poor appetite, which were similar to the findings reported by the SHARP trial [27]. No AE-related death was found in the two groups. However, the incidence of AEs was significantly lower in the TACE-Sorafenib group than that in the TACE-Apatinib group (χ^2^ = 11.72, *P* = 0.001). After the dose adjustment according to the grade 3–4 AEs, the incidence of AEs was not significantly different between the two groups (χ^2^ = 2.09, *P* = 0.148), while the incidence of AEs in the TACE-Apatinib group was significantly lower after the dose adjustment than before the dose adjustment (χ^2^ = 11.72, *P* < 0.001). The higher incidence of AEs in the TACE-Apatinib group than that in the TACE-Sorafenib group in patients with intermediate or advanced HCC could be associated with the higher activities of Apatinib [36], and the AEs could be associated with the inhibition of VEGF signaling pathway.

Subgroup analysis showed that the survival time of patients was not significantly different between the TACE-Sorafenib and TACE-Apatinib groups when the regular doses were administered (22.59 vs. 19.75, *P* = 0.118), and the survival time was not significantly different between the two groups after the dose reduction (18.02 vs. 16.86, *P* = 0.242). The survival time of patients treated with regular doses of drugs was significantly longer than that of treated with reduced doses of drugs (Apatinib, 19.75 vs. 16.86, *P* = 0.005; Sorafenib, 22.59 vs. 18.02, *P* < 0.001), suggesting that both Sorafenib and Apatinib possess certain treatment efficacies for patients with intermediate or advanced HCC. However, treatments are commonly limited by serious AEs, which could make it impossible to perform standard treatments, and consequently deprive patients from significant treatment benefits. Concerning a significantly higher incidence of AEs and patients requiring dose adjustment in the TACE-Apatinib group than that in the TACE-Sorafenib group, we speculated that the causes of the significantly longer OS in the TACE-Sorafenib group than the TACE-Apatinib group could be associated with the higher tolerance of Sorafenib, which allowed to the majority of patients to receive a standard treatment.

## Conclusion

In summary, TACE plus either Sorafenib or Apatinib could effectively treat patients with intermediate or advanced HCC, and the safety of TACE plus Sorafenib was higher. As the ORR of TACE plus Apatinib was higher, Apatinib treatment could exert more potential benefits, especially for improving the ORR of advanced HCC patients, and therefore provide opportunity for patients to receive rapid transformational therapy. However, This study also has certain shortcomings. First, the number of patients enrolled is not large enough and belongs to a retrospective study, further prospective, multi-center, randomized controlled studies are required to verify our findings.

## Data Availability

Not applicable.

## Abbreviations

AEs: Adverse events
VEGF: Vascular endothelial growth factor
MMP: Matrix metallopeptidase
MMPs: Matrix metalloproteinases
VEGFR: Vascular endothelial growth factor receptor
PDGFR: Platelet-derived growth factor receptor
OS: Overall survival
KDR: Kinase insert domain receptor
ORR: Objective response rate
DCR: Disease control rate
PVA: Polyviny alcohol
AFP: Alpha-fetoprotein
mRECIST: Modified Response Evaluation Criteria in Solid Tumors
MRI: Magnetic Resonance Imaging
CTCAE: Common Terminology Criteria Adverse Events
PSM: Propensity score matching
PFS: Progression-free survival
HCC: Hepatocellular carcinoma
TACE: Transcatheter arterial chemoembolization
PSM: Propensity score matching
ECOG PS: Eastern Cooperative Oncology Group Performance Status
MVI: Microvascular invasion
